# Interventions to improve vitamin D status in at-risk ethnic groups during pregnancy and early childhood: a systematic review

**DOI:** 10.1017/S1368980021000756

**Published:** 2021-08

**Authors:** Nuttan K Tanna, Emma C Alexander, Charlotte Lee, Monica Lakhanpaul, Rickin M Popat, Pamela Almeida-Meza, Alice Tuck, Logan Manikam, Mitch Blair

**Affiliations:** 1 London North West University Healthcare NHS Trust, Northwick Park Hospital, Watford Road, Harrow, London HA1 3UJ, UK; 2 Imperial College London, Department of Primary Care and Public Health, Level 2, Faculty Building, South Kensignton Campus, London SW7 2AZ, UK; 3 UCL Great Ormond Street, Institute of Child Health, London, UK; 4 Whittington Health NHS Trust, London, UK; 5 Royal Free London NHS Foundation Trust, Barnet Hospital, London, UK; 6 UCL Institute of Epidemiology & Health Care, London, UK; 7 Aceso Global Health Consultants Ltd, London, UK

**Keywords:** Vitamin D, Ethnic minorities, Children, At risk, Optimisation, Interventions

## Abstract

**Objective:**

To systematically review the literature with the primary aim of identifying behavioural interventions to improve vitamin D stores in children from at-risk ethnic groups.

**Design:**

Review based on Preferred Reporting Items for Systematic Reviews and Meta-Analyses (PRISMA) guidelines. PROSPERO registration number: CRD42017080932. Health Behaviour Model and Behaviour Change Wheel framework constructs used to underpin evaluation of interventions. Methodological quality evaluated using Cochrane Risk of Bias, Cochrane ROBINS-I and NHLBI tools.

**Setting:**

Databases Cochrane Library, MEDLINE, EMBASE, CINAHL with secondary search of Google Scholar. No country limits set. Papers between January 1990 and February 2018, published in English included. Anticipating study heterogeneity, outcome measures not pre-specified and identified from individual full papers. Updated literature search November 2020.

**Participants:**

Patient or population including pregnant women, newborns and children aged under 18 years, from Asian or African ethnic groups.

**Results:**

Of 10 690 articles screened, 298 underwent full-text review, with 24 ultimately included for data extraction. All identified studies conducted a vitamin D pharmacological supplementation intervention, with two also incorporating a behavioural intervention strategy. No study explicitly defined a primary aim of evaluating a behavioural intervention, undertaken to study its effect on vitamin D supplement uptake.

**Conclusions:**

There is a need to address the paucity of data in ethnic at-risk children on how behavioural interventions ideally developed and co-produced with the community under study, affect and help improve vitamin D uptake, within the antenatal and pregnancy phase as well as during childhood.

Vitamin D deficiency is a global health problem affecting over 1 billion people worldwide^(1)^. Vitamin D is an essential nutrient that contributes to bone health, and deficiency status increases the risk of rickets, osteomalacia and osteoporosis^(2–4)^. Pregnant women and their breast-fed neonates as well as older children are at particular risk for vitamin D deficiency, as are Black and ethnic minority groups^(1)^. Hypovitaminosis D^(5)^ is common in pregnant women (up to 50 %) and their breast-fed infants (up to 56 %^(6)^) and has been reported in 64 % Middle Eastern women, 58 % Black women and 47 % Asian women, compared to only 13 % of Caucasian women^(7)^. Short-term implications include lower neonate birth weight, length and head circumference^(6,8–10)^ and skeletal outcomes such as muscle pain and fractures^(11)^. Hypo-calcaemic seizures, rickets and cardiomyopathy have been reported in children under 5 years^(2,12)^. Deficiency in childhood is associated with obesity and metabolic syndrome^(13)^ and is a significant risk factor for dental caries^(10,14,15)^.

The major cause of vitamin D deficiency is low transmission or reduced penetration of solar ultra-violet B radiation, affecting the cutaneous synthesis of vitamin D. Although found naturally in some foods such as oily fish, red meat, liver and egg yolks and fortified foods (infant formula, breakfast cereals and fat spreads), less than 10 % of vitamin D stores come from individual diets^(3,16)^. Often, the main source of dietary vitamin D is in the form of supplements. In women of child-bearing age, supplementation rates range between 12 and 27 %^(17)^, and data indicate that 63 % women of reproductive age are vitamin D-deficient^(18)^. The Royal College of Paediatrics and Child Health and the British Paediatric Surveillance Unit recently ascertained the national incidence of nutritional rickets in children under the age of 16 years^(2)^. Black and South Asian children had a 10-fold and 5-fold greater incidence of nutritional rickets, respectively, compared with other ethnic groups under 5 years of age.

Despite current national policy and guidance to ensure supplementation in high-risk groups^(1,3,19,20)^, uptake is not consistent and a deeper understanding of facilitators and barriers to improve uptake is required. Behavioural interventions have shown some promise in contributing to the prevention, management and treatment of various other non-communicable diseases; obesity, diabetes, chronic pain, asthma and emotional difficulties^(21)^. In this paper, we refer to ethnic minority groups, accepting that the term is often used interchangeably with race^(22)^. Race is a social construct, usually identified based on a combination of physical, cultural and behavioural attributes, whereas ethnicity, self-identified by an individual, encompasses aspects such as nationality, culture, language and religion^(23)^. There are known structural barriers that Black, Asian and minority ethnic (BAME) groups face when accessing healthcare or being involved in clinical research^(24–26)^. Lack of exposure to health promotion messages and economic disadvantage are factors contributing to health inequalities. Considering these inter-related complexities, it has been recognised that interventions that are developed with the community of interest and which are culturally sensitive and tailored may be more effective^(24)^. Our teams have a record of working with at-risk communities. With time, we have become increasingly aware of the need to understand how behavioural interventions may help at-risk communities to comply with public health advice, including that for vitamin D supplementation. Acknowledging this background, we undertook a systematic review to evaluate behavioural interventions implemented to improve or optimise vitamin D stores in children from at-risk ethnic groups.

## Methods

Our systematic review, performed based on the Preferred Reporting Items for Systematic Reviews and Meta-Analyses (PRISMA) guidelines^(27)^, is registered within the PROSPERO database, CRD42017080932.

Identified interventions were planned to be evaluated using the Health Behaviour Model (HBM)^(28)^. The HBM^(28)^ attempts to explain and predict health-related behaviours across four constructs: (i) perceived susceptibility (i.e. affect with known risk of deficiency); (ii) perceived severity (i.e. affect with known consequences/health outcomes of deficiency); (iii) perceived benefits (defined here as promoters for optimal vitamin D status, i.e. sufficient sun exposure, supplementation, health information access); and (iv) perceived barriers (i.e. sufficient sun exposure, supplementation, health information access)^(29)^.

Classification of behavioural interventions was to be based on the nine categories within the Behaviour Change Wheel framework^(30)^: (1) education (e.g. increasing knowledge or understanding around benefits of supplementation/sun exposure or consequences of deficiency); (2) persuasion (e.g. reminder to supplement); (3) incentivisation (e.g. expectation of reward); (4) coercion (e.g. expectation of punishment, cost or fine); (5) training (e.g. how to take/administer supplements or drops to children); (6) restriction; (7) environmental restructuring (e.g. shared decision-making with General Practitioners (GP)/health professionals); (8) modelling (e.g. providing examples); and (9) enablement (e.g. reducing barriers).

### Search question

The PICO acronym, an established model for aiding systematic reviews, guided all elements of our research question:

1. Patient or Population: children under 18 years of age from an Asian or African ethnic group or race, including pregnant women and their newborns.
2. Intervention: to optimise vitamin D status (thresholds for optimisation as defined by research group), with primary focus on behavioural studies.
3. Comparator: all controls.
4. Outcomes: improvement in vitamin D status (no pre-specified definition).

### Data sources

The following databases were searched and verified: Cochrane Library, MEDLINE, EMBASE, CINAHL with limited secondary search on Google Scholar, in order to identify relevant studies. No country limits set. Only articles written in English published after 1990 were included. Searches were conducted in February 2018, with a limited updated search in November 2020.

### Search strategy

The search strategy included terms for ‘vitamin D’ and ‘ethnic’ and terms specifying all major subgroups. Details are as follows:

Term 1: Vitamin D

- Vitamin D * OR ricket* OR osteomalacia

Term 2: Ethnic

- Ethnic group* OR Asia* OR Africa* OR emigrant* OR immigrants

Search string:

- (Vitamin D * OR ricket* OR osteomalacia) AND (ethnic group* OR Africa* OR Asia* OR emigrant* OR immigrant*)

A selective search strategy focusing on identifying behavioural interventions was used for updated review of abstracts February 2018–November 2020 (Additional File 1).

### Eligibility criteria

We classified Asian ethnic groups as individuals of central, east, south, south-east, and western Asian origin and African as individuals of east, north, south, and western and south of the Sahara origin. Studies were included if they met the PICO inclusion criteria above, were published in English or with translation available, and were randomised controlled trials (RCT), quasi-RCT and non-RCT (before-after studies).

We excluded purely observational studies, non-English language, or no English version available, full-text not available, or studies classified as dissertations/abstracts/conference pieces/editorial letter or review. We also excluded studies with no or non-extractable ethnic/demographic or vitamin D data and those focusing on adult populations.

### Study selection and data extraction

Two reviewers (EA and CL) shared screening of titles and abstracts. Shortlisted abstracts underwent full-text review by two researchers (PA and AT), with conflicts resolved by a third reviewer (CL). For each study of interventions, data were extracted and classified (AT and PA) and checked (EA and NT) for publication year, characteristics of study population (sample size, mean age, ethnicity, gender and age), study design, available vitamin D data, supplementation data and intervention measured. For the updated search in November 2020, in line with our primary aim, we searched only for studies with an explicitly defined behavioural intervention. Titles and abstracts were screened by two reviewers (RP and NT) and confirmed with third reviewer (EA).

### Risk of bias assessment

Methodological quality of the studies was assessed using the Cochrane Risk of Bias-2^(31)^ assessment tool for RCT (nineteen studies), the Cochrane ROBINS-I^(32)^ tool for non-randomised studies of interventions (one study) and the NHLBI^(33)^ tools for Observational Cohort and Cross-Sectional Studies (one study) or Before-After (Pre-Post) Studies with No Control Group (three studies). The studies were quality-assessed by EA, with ratings reviewed by NT, points of uncertainty discussed and final ratings agreed with NT (*n* 5; 22 %). Studies with a high or critical risk of bias were included but with quality ratings highlighted within the results section in order to contextualise findings.

### Results synthesis

In view of the heterogeneity of studies identified in terms of methods, participants, vitamin D thresholds used, the interventions and outcomes, a narrative approach to synthesis was used following guidance developed by the University of York Centre for Reviews and Dissemination and the Economic and Social Research Council^(34,35)^.

## Results

### Study selection

**Fig. 1:**
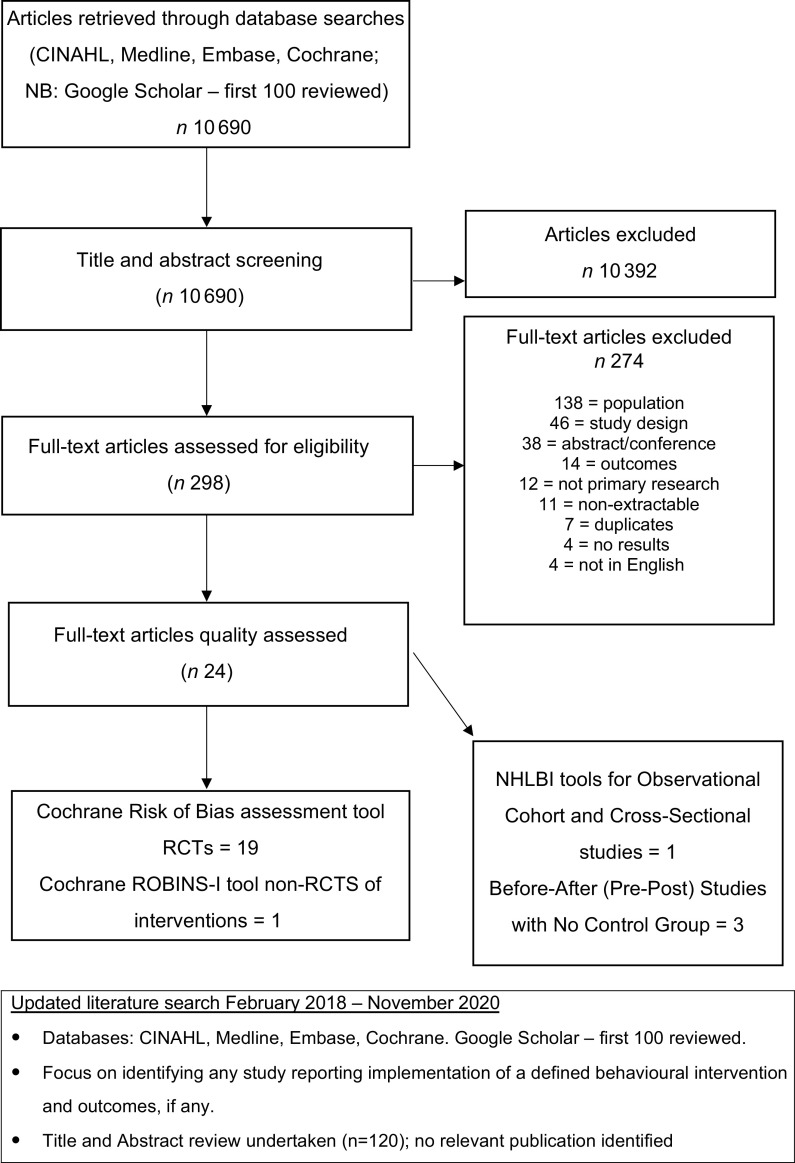
Study flow diagram. RCT, randomised controlled trial

Initially, 10 690 articles were identified. Title and abstract screening returned 298 potential articles. After full-text review, 274 articles were excluded, and 24 intervention studies (including 2 with behavioural components) were included. No further behavioural studies were identified from the updated literature search in November 2020 (see Fig. 1).

### Study characteristics

The majority^(11)^ of studies included were conducted in the USA, with two further studies each in Australia, Canada, and Mongolia. One study was undertaken in each of the following: UK, Norway, Denmark, Pakistan, India, Nigeria, and Turkey. Table 1 summarises these papers.

**Table 1:** Studies included in systematic review

Reference	Country – latitude if stated	Design	Population	Sample size	Age (years)	Intervention – pharmacological/behavioural	Vitamin D measure (sufficiency/insufficiency/deficiency threshold used).	Primary findings
Abrams *et al.* (2013)^(36)^	USA – sunny latitude	8-week double-blind RCT	Healthy pre-pubertal; African American (12), Hispanic (23), Asian (1) and White (27)	64 (one lost to follow-up)	4–9	Supplementation with 1000 iu/d for 8 weeks.	Serum 25(OH)D (baseline 25(OH)D concentrations: low (20 ng/ml) 50 nmol/l; middle (20–32 ng/ml) 50–80 nmol/l; high (32 ng/ml)) 80 nmol/l).	Vitamin D_3_ supplementation with 1000 iu taken daily increased 25(OH)D levels and decreased parathyroid hormone in children, whose average vitamin D intake was below Institute of Medicine dietary recommendation; but with no significant effect of change in vitamin D_3_ levels on Ca absorption, as after 8 weeks, with baseline values used as a covariate, no differences were seen in fractional or total Ca absorption based on supplementation group (*P* = 0·75 and 0·36, respectively).
Andersen *et al.* 2008^(56)^	Denmark – Copenhagen area, (latitude 550N)	1 year-double-blind RCT	Adolescent girls Pakistani origin NB: women (18·1–52·7 years) and men (17·9–63·5 years).	21 girls Overall dropout rate 19 % NB: 62 women and 65 men.	(10·1–14·7 years)	Two doses of vitamin D_3_ (10 and 20 mcg/d).	Serum 25(OH)D Main study endpoints: serum 25(OH)D, S-iPTH, bone turnover markers and markers of bone mass (whole-body and lumbar spine bone area (BA), BMD and bone mineral content (BMC)).	Difficult to comment on any significant difference in serum vitamin 25(OH)D_3_ levels in the group of girls at end of study due to the low number recruited and high dropout rate. NB: Supplementation with 10 and 20 mcg vitamin D_3_/d increased serum 25(OH)D levels similarly in vitamin D-deficient Pakistani women (fourfold); 10 mcg increased serum-25(OH)D concentrations twofold and 20 mcg threefold in Pakistani men. Serum 25(OH)D concentrations increased at 6 months and were stable thereafter.
Dong *et al.* (2010)^(50)^	USA – sunny latitude	16-week open-label investigator-blinded RCT	Healthy African American	49	14–18	Supplementation with 2000 iu/d compared with 400 iu/d for control group.	Serum 25(OH)D (sufficiency: 25(OH)D level of at least 75 nmol/l. Insufficiency: more than 50 but less than 75 nmol/l. Deficiency: under 50 nmol/l).	Study group reached significantly higher 25(OH)D levels at 8 and 16 weeks. The 2000 iu group achieved 25(OH)D levels of 85·7 +/− 30·1 nmol/l at 16 weeks from 33·5 +/− 9·9 nmol/l at baseline; *P* < 0·001. Daily 2000 iu vitamin D supplementation may be effective in optimising vitamin D status and counteracting the progression of aortic stiffness in Black youth. Plasma vitamin 25(OH)D levels in response to 2000 iu/d supplementation negatively modulated by adiposity
Dougherty 2015^(51)^	Philadelphia, USA	12-week RCT	African American children and young adults with SCD.	21 with and 23 without SCD-SS	5–20 years old	To assess safety and efficacy of two oral daily doses (4000 v. 7000 iu) of cholecalciferol (vitamin D_3_).	Serum 25(OH)D With suboptimal vitamin D status (<32 ng/ml).	After 12-week supplementation, both vitamin D_3_ doses were safe (no hypercalcaemia) and well tolerated. For both groups, deficient (< 20 ng/ml) vitamin D status was eliminated only in those receiving 7000 iu/d (*P* < 0·05).
Ganmaa *et al.* (2008)^(39)^	Mongolia (East Asia) – Northern latitude	Observation/pilot feasibility study	Mongolian	46	9–11	Supplementation with three 236-ml tetra pack boxes of conventional UHT-processed fortified whole milk daily (100 iu vitamin D/serving).	Serum 25(OH)D	After 1 month of drinking vitamin D fortified milk, vitamin 25(OH)D levels were improved.
Ganmaa 2017^(57)^	Mongolia (East Asia) – Northern latitude	(i) 6-month RCT (ii) 7-week cluster-randomised classroom-based	Urban school age children	(i) 113 (ii) 235	(i) 12–15 years (ii) 9–11 years	(i) 800 iu vitamin D daily. (ii) 710-ml whole milk fortified with 310 iu vitamin D, daily.	Serum 25(OH)D Deficiency defined as <20 ng/ml.	Correction of baseline vitamin D deficiency in children with vitamin D 800 iu supplement daily over 6 months increased growth.
Green *et al.* (2015)^(37)^	Canada	Observation	Asian (28) and White (37)	65	0·2–0·4	Fifty-eight (89 %) breast-fed term infants had been given a vitamin D supplement, in most cases providing 10 mcg/dose.	Serum 25(OH)D	Mean 25(OH)D levels of the infants = 31 ng/ml, 95 % CI, 28–34 ng/ml. Infants who received 10 mcg vitamin D achieved significantly higher 25(OH)D concentrations than those who received less; mean 25(OH)D 9·4 ng/ml higher; *P* = 0·003. Maternal vitamin 25(OH)D, season, skin colour and ethnicity were not significant determinants of infant vitamin 25(OH)D levels.
Hanson *et al.* (2011)^(46)^	Canada	RCT	Infants receiving formula feedings during intensive care hospitalisation. White/non-White infants (African American)	52	Newborn; infants 32 weeks gestational age to discharge	Supplemented group received 400 iu/d vitamin D_3_ in addition to formula feedings; control group received matching placebo.	Serum 25(OH)D Infants measured from cord blood, blood serum test every 7 d, and at discharge. Intact parathyroid hormone was measured at discharge.	In newborn intensive care unit hospitalised infants, vitamin D_3_ supplementation of 400 iu/d increased mean vitamin 25(OH)D levels. White infants had statistically significant higher mean 25(OH)D levels at discharge compared with non-White infants (*P* = 0·0003). Mean supplemented group 23·1 +/− 7 ng/ml (57·66 +/− 17·47 nmol/l); Mean non-supplemented group 17·8 +/− 4·7 mg/ml (44·43 +/− 11·3 nmol/l (*P* = 0·007)
Hollis *et al.* (2011)^(48)^	USA – 32° North	Single-centre RCT	Women with singleton pregnancies at 12–16 weeks gestation stratified by self-defined race/ethnic group Caucasian; 115 African American; 98 Hispanic; 137 completed study	350	Pregnancy/newborn	Vitamin D supplementation daily until delivery; doses 400 iu daily; 2000 iu daily; 4000 iu daily. NB: Tablet concentration verified by independent company every 6 months.	Primary outcome maternal/neonatal circulating 25(OH)D at delivery; secondary outcomes 25(OH)D > or = 80 nmol/l achieved?; 25(OH)D concentration required to achieve maximal 1,25(OH)D production. Deficiency defined as total circulating 25(OH)D < 50 nmol/l (20 ng/ml), insufficiency as ≥50 to <80 nmol/l (≥20 to <32 ng/ml), and sufficiency as ≥80 nmol/l (≥32 ng/ml).	Mean vitamin 25(OH)D levels by group at delivery and 1 month before delivery was significantly different (*P* < 0·0001). 4000 iu daily dose in pregnancy is safe and most effective in achieving sufficiency in all women regardless of race (*P* < 0·0001).
Hollis *et al.* (2015)^(43)^	Rochester, New York, USA	6-month RCT	Mother–infant pairs; Maternal race/ethnicity: Black (African American)/Hispanic/White	334	Newborn/breast-feeding 4–6 weeks old, with planned breast-feeding for 6 months	Maternal vitamin D_3_ supplementation with 6400 iu/d v. maternal and infant supplementation of 400 iu d.	Maternal serum/neonatal serum 25(OH)D. Vitamin D deficiency < 50 nmol/l.	Compared with 400 iu vitamin D_3_/d, 6400 iu /d was safe; with significant increase in maternal vitamin 25(OH)D from baseline (*P* < 0·0001). Vitamin D deficiency in breast-fed infants affected by race. African American mothers and infants had substantially lower circulating 25(OH)D levels; several minority infants had severe vitamin D deficiency (2·5 nmol/l 25(OH)D) after 1 month of breast-feeding.
Hossain *et al.* 2014^(47)^	Pakistan, South Asia	Single-centre open-label RCT	Pakistani pregnant women and neonates	193	Pregnancy/newborn, within 48 h of birth	4000 iu vitamin D daily from week 20 to delivery v. routine care (200 mg ferrous sulphate and 600 mg Ca).	Serum 25(OH)D Cord blood or neonatal serum levels. Maternal serum at baseline and delivery.	Maternal vitamin D supplementation improved maternal and neonatal vitamin D status. Maternal 25(OH)D levels increased in treated group 18·3 +/− 11 ng/dl v. 8·82 +/− 11·84 ng/dl; *P* = 0·001. Routine care group 25(OH)D levels 6·9 +/− 7·0 ng/dl v. 6·32 +/− 3·97 ng/dl; *P* = 0·6. There was a positive correlation between maternal and neonatal 25(OH) vitamin D levels (r = 0·83; *P* = 0·001).
Lewis *et al.* (2013)^(54)^	USA – latitudes 34°N and 40°N),	RCT – multisite triple-masked trial	Black and White	323	9–13	Children received 1 of 5 oral vitamin D_3_ doses (0, 400, 1000, 2000 and 4000 iu/d) and were sampled over 12 weeks.	Serum 25(OH)D Serum 1,25(OH)2D	Mean baseline 25(OH)D for entire sample 70 nmol/l. Increases in 25(OH)D levels depended on dose taken, with 12-week changes ranging from −10 nmol/l for placebo to 76 nmol/l for 4000 iu. Larger vitamin 25(OH)D gains observed for Whites compared with Black children at highest dose (*P* < 0·01).
Madar *et al.* (2009)^(42)^	Norway	RCT	Pakistani, Turkish and Somali	51	7-week-old	7-week supplementation of 400 iu of vitamin D_2_ and tailor-made information about vitamin D and its sources.	Serum 25(OH)D	Vitamin D supplementation provided free of charge together with translated information brochures significantly increased vitamin serum 25(OH)D concentrations when compared to a control group receiving usual care.
Mondal *et al.* (2014)^(44)^	India	RCT	Indian children with nutritional rickets.	71	6 months to 5 years	Single intramuscular dose of 600 000 iu of vitamin D or 10 weeks of 60 000 iu of vitamin D taken weekly for 10 weeks. 12-week follow-up.	Serum 25(OH)D	No difference found in efficacy of the two regimens on comparing diagnostic parameters for rickets. Both treatments were safe and effective. No difference in mean serum 25(OH)D levels in both groups at baseline and 12 weeks (*P* = 0·887); Increase in serum 25(OH)D in both groups statistically significant; *P* < 0·001 in both groups at 12 weeks v. baseline. No significant difference between groups on comparing proportion of children with biochemical features suggestive of rickets at any point of the study (*P* > 0·05), except at 4 week follow-up, there were more cases of hypocalceamia in oral group (*P* = 0·039).
Mutlu 2011^(38)^	Turkey (Middle East/West Asia)	Observational cohort cross-sectional study to evaluate efficacy of nationwide prevention programme	Turkish infants, recalled as part of national screening for congenital hypothyroidism; Information regarding age at start of supplementation, the dosage and compliance obtained from mothers with face-to-face interview.	85	16·5 +/− 20·7 (3–120) d	Babies had been provided with free vitamin D (cholecalciferol) solution and recommended to receive 400 iu (3 drops) daily.	Serum 25(OH)D Mean 25-(OH)D level was 42,5 ± 25,8 (median: 38·3) ng/ml.	Taking 400 iu/d of vitamin D seems adequate to prevent vitamin D deficiency.
Rajakumar *et al.* (2005)^(41)^	Philadelphia, USA	Non-randomised pre-post comparison	African American; at-risk vitamin D sample	Total 41: 27 males and 14 females	6–10	4-week 400 iu vitamin D supplementation.	Serum 25(OH)D 1,25(OH)2D (i) 12–20 ng/ml essential for bone health in children. (ii) Deficiency: less than 10 ng/ml. Insufficiency: 10–20 ng/ml based on definition for adults.	Vitamin D insufficiency in approximately half of participants at baseline. Although with supplementation vitamin D levels increased significantly*, vitamin D insufficiency persisted in 18 % of experimental group. *Significant incremental increase in 25(OH)D levels found only among vitamin D insufficient children (15·4 +/− 3·2 ng/ml to 23 +/− 6·4, *P* < 0·001 v. 32·1 +/− 8·1 ng/ml to 31·8 +/− 5·8 ng/ml, *P* = 0·79.
Rajakumar *et al.* (2008)^(40)^	Philadelphia, USA	Non-randomised pre-post comparison	African American	Total 41: 21 obese and 20 non-obese	6–10	4-week 400 iu/d vitamin D supplementation.	Serum 25(OH)D Deficiency: less than 20 ng/ml. Insufficiency: 21–29 ng/ml.	Vitamin D deficiency common among obese and non-obese pre-adolescent African American children. Vitamin D 400 iu daily dose for 1 month is not enough to raise blood levels to 30 ng/ml. Vitamin D deficiency in 12/21 (57 %) obese v. 8/20 (40 %) non-obese at baseline (*P* = 0·35); persisted in 5/21 (24 %) obese v. 2/18 (11 %) non-obese (*P* = 0·42) after treatment.
Rajakumar *et al.* (2015)^(49)^	Philadelphia, USA	RCT	African American (84) and White (73)	157	8–14	6 months supplementation with vitamin D_3_ 1000 iu/d	Serum 25(OH)D	Mean circulating levels of vitamin D were higher in supplemented group v. placebo group. Effect of vitamin D_3_ supplementation varied by race and was found to be more effective and significant in Black children*. *Levels at 2 months 26·4 +/− 8·1 v. 18·9 +/− 8·1 ng/ml; *P* < 0·001 and at 6 months 26·7 +/− 7·6 v. 22·4 +/− 7·3; *P* = 0·003. Lower baseline 25(OH)D levels associated with greater change at 2 months overall (r = −0·49; *P* < 0·001 but not in White children (r = -0·0005; *P* = 1·0).
Rajakumar *et al.* (2016)^(52)^	Philadelphia, USA	RCT	African American (52) and White (44)	96	8–14	Supplemented for 6 months with 1000 iu/d vitamin D_3_	Serum 25(OH)D	At baseline, Black children had lower mean 25(OH)D levels (*P* < 0·0001) and higher Ca levels (*P* = 0·001) than White children. With intervention, the within-group change *P*-value was <0·0001 for treated v. 0·041 for placebo group. Vitamin D intake required in order to maintain 25(OH)D levels at 20 ng/ml in 97·5 % of Black and White children, adjusting for race and pubertal status, was three times higher than (current USA) recommended daily allowance of 600 iu/d.
Rodda *et al.* (2015)^(45)^	Australia, latitude 38 degrees south	RCT	Vitamin D-deficient pregnant women and their newborns. Dark skin or veiled: 84 % in treated group v. 97 % control group.	78	Pregnancy; newborn Treatment 12–16 weeks’ gestation to delivery when maternal and neonate vitamin D levels tested	Supplementation with 2000–4000 iu/d until 28-week gestation	Serum 25(OH)D	Recruited women with singleton pregnancies with serum 25(OH)D levels less than 75 nmol/l, defined as deficiency or insufficiency, at first antenatal appointment at 12–16 weeks gestation. Umbilical cord serum 25(OH)D concentration at delivery was higher in neonates of the treatment group mothers compared with neonates of control group mothers (81 nmol/l (range 70–91) v. 42 nmol/l (range 34–50), CI 95 %; *P* < 0·0001). Strong positive correlation between maternal and umbilical serum 25(OH)D levels at delivery; spearman rank correlation coefficient 0·88; *P* < 0·0001
Sacheck *et al.* (2017)^(53)^	USA	RCT	Black, Hispanic, White and Asian.	604	8–15	Supplemented with vitamin D_3_ at 600, 1000 or 2000 iu/d for 6 months	Serum 25(OH)D	Serum vitamin 25(OH)D levels increased over 6 months in all three dose groups. The 2000 iu/d group achieved a higher vitamin D concentration than the other two dose groups (33·1 v. 26·3 and 27·5 ng/ml; *P* < 0·001).
Talib *et al.* (2016)^(55)^	USA, New York latitude 40·8 °NE)	RCT	Adolescents with vitamin D deficiency Hispanic, Black, White and Asian	122	13–20 years; mean age 16·6 +/− 2·2	Supplemented for 8 weeks with either 50 000 iu/week, 5000 iu/d or 1000 iu/d.	Serum 25(OH)D	Adolescents require 8 weeks of high-dose cholecalciferol (at least 5000 iu/d) to correct vitamin D deficiency. Obese adolescents have poorer response to treatment (13·7 +/− 10·7 v. 21·9 +/− 16·9 ng/ml; *P* < 0·001). Mean change in 25(OH)D levels post-treatment: 24·9 +/− 15·1–50 000 iu group v. 21·0 +/− 15·2–5000 iu group v. 6·2 +/− 6·5 ng/ml–1000 iu respectively; *P* < 0·001.
Thacher *et al.* (1999)^(58)^	Nigeria, Africa	RCT	Nigerian children with rickets or bone deformities.	109	1–14 years	Supplemented for 24 weeks with either vitamin D 600 000 iu intramuscularly, calcium 1000 mg/d or a combination of vitamin D and calcium.	Serum 25(OH)D	Calcium supplementation with or without vitamin D was more effective than supplementation with vitamin D alone in healing active rickets (*P* < 0·001). Daily dietary calcium intake was low in both children with rickets and control group children (median 203 mg and 196 mg, respectively; *P* −0·64).
Yu *et al.* 2009^(7)^	London, UK	Prospective RCT,	Indian Asian, Middle Eastern, Black and Caucasian	180	Study arms 27 weeks’ gestation to delivery	Study arms: single-dose 200 000 iu/800 iu daily/no treatment	Mothers – serum 25(OH)D. Babies – cord 25(OH)D. Thresholds: sufficient > 50 nmol/l; insufficient 25–50 nmol/l; deficiency < 25 nmol/l.	Final maternal 25(OH)D levels supplemented v. no treatment; daily dose median 42 (31–76) v. 27 (27–39) nmol/l; *P* < 0·0001. Cord levels significantly higher with supplementation; daily dose median 25 (18–34) v. 17 (14–22) nmol/l in no-treatment group; *P* = 0·001. Single or daily dose improved serum vitamin D levels significantly. Only small percentage of women (30 %) and babies (8 %) who were given supplements were vitamin D-sufficient

RCT, randomised controlled trial; SCD, sickle cell disease; UHT, ultra-high-temperature.

*Serum/plasma concentration of 25-hydroxyvitamin-D (25(OH)D), the major circulating metabolite of vitamin D, is expressed as nanomoles per litre (nmol/l) or nanograms per millilitre (ng/ml); 2·5 nmol/l is equivalent to 1 ng/ml. Due to differences in molecular mass and the amount required to prevent rickets, there is no absolute agreement on conversion of ng to nmol for 25(OH)D_2_ and 25(OH)D_3_. Inconsistencies relating to these measurements need consideration when interpreting studies comparing vitamin D_2_ and D_3_^(70)^.

With reference to quality appraisal, nineteen studies were appraised using Cochrane Risk of Bias for RCT. Of these, one^(36)^ had a low risk of bias and the remaining eighteen had some concerns. One study^(37)^, appraised with the ROBINS-I tool, was coded as having a critical risk of bias due to confounding. Of the NHLBI appraised studies, two were rated as fair^(38,39)^ and two as good^(40,41)^ (see Tables 2–5).

**Table 2:** Cochrane risk of bias for randomised controlled trials (RCT)^(31)^

	Risk of bias arising from the randomisation process	Risk of bias due to deviations from the intended interventions (effect of assignment to intervention)	Risk of bias due to deviations from the intended interventions (effect of adhering to intervention)	Missing outcome data	Risk of bias in measurement of the outcome	Risk of bias in selection of the reported result	Overall risk of bias
Abrams *et al.* (2013)^(36)^	Low	Low	Low	Low	Low	Low	Low
Anderson *et al.* 2008^(56)^	Some concerns	Low	Low	Some concerns	Low	Some concerns	Some concerns
Dong *et al.* (2010)^(50)^	Low	Some concerns	Some concerns	Some concerns	Low	Some concerns	Some concerns
Dougherty 2015^(51)^	Some concerns	Low	Some concerns	Some concerns	Low	Some concerns	Some concerns
Ganmaa 2017^(57)^	Some concerns	Low	Some concerns	Low	Low	Low	Some concerns
Hanson *et al.* (2011)^(46)^	Low	Some concerns	Some concerns	Low	Low	Low	Some concerns
Hollis *et al.* (2011)^(48)^	Low	Low	Low	Some concerns	Low	Low	Some concerns
Hollis *et al.* (2015)^(43)^	Low	Some concerns	Low	Some concerns	Low	Low	Some concerns
Hossain N *et al.* 2014^(47)^	Low	Low	Low	Low	Some concerns	Some concerns	Some concerns
Lewis *et al.* (2013)^(54)^	Low	Low	Some concerns	Low	Low	Some concerns	Some concerns
Madar *et al.* (2009)^(42)^	Some concerns	Low	Some concerns	Some concerns	Some concerns	Low	Some concerns
Mondal *et al.* (2014)^(44)^	Some concerns	Low	Low	Some concerns	Some concerns	Some concerns	Some concerns
Rajakumar *et al.* (2015)^(49)^	Low	Low	Low	Some concerns	Low	Low	Some concerns
Rajakumar *et al.* (2016)^(52)^	Low	Low	Low	Some concerns	Low	Low	Some concerns
Rodda *et al.* (2015)^(45)^	Low	Some concerns	Low	Some concerns	Some concerns	Low	Some concerns
Sacheck *et al.* (2017)^(53)^	Low	Low	Low	Some concerns	Low	Low	Some concerns
Talib *et al.* (2016)^(55)^	Low	Some concerns	Low	Some concerns	Low	Low	Some concerns
Thatcher *et al.* (1999)^(58)^	Some concerns	Low	Low	Low	Low	Some concerns	Some concerns
Yu *et al.* 2009^(7)^	Some concerns	Low	Low	Low	Some concerns	Low	Some concerns

**Table 3:** Cochrane ROBINS-I^(32)^

	Bias due to confounding (low/moderate/serious/critical/no info (NI))	Bias in selection of participants into the study (low/moderate/serious/critical/NI)	Bias in classification of interventions (low/moderate/serious/critical/NI)	Bias due to deviations from intended interventions (low/moderate/serious/critical/NI)	Bias due to missing data (low/moderate/serious/critical/NI)	Bias in measurement of outcomes (low/moderate/serious/critical/NI)	Bias in selection of the reported result (low/moderate/serious/critical/NI)	Overall bias
Green *et al.* (2015)^(37)^	Critical	Moderate	Low	Moderate	No Info	Moderate	Moderate	Critical

**Table 4:** Quality assessment tool for observational cohort and cross-sectional studies^(33)^

	Was the research question or objective in this paper clearly stated?	Was the study population clearly specified and defined?	Was the participation rate of eligible persons at least 50%?	Were all the subjects selected or recruited from the same or similar populations (including the same time period)? Were inclusion and exclusion criteria for being in the study pre-specified and applied uniformly to all participants?	Was a sample size justification, power description, or variance and effect estimates provided?	For the analyses in this paper, were the exposure(s) of interest measured prior to the outcome(s) being measured?	Was the time frame sufficient so that one could reasonably expect to see an association between exposure and outcome if it existed?	For exposures that can vary in amount or level, did the study examine different levels of the exposure as related to the outcome (e.g. categories of exposure, or exposure measured as continuous variable)?	Were the exposure measures (independent variables) clearly defined, valid, reliable, and implemented consistently across all study participants?	Was the exposure(s) assessed more than once over time?	Were the outcome measures (dependent variables) clearly defined, valid, reliable, and implemented consistently across all study participants?	Were the outcome assessors blinded to the exposure status of participants?	Was loss to follow-up after baseline 20% or less?	Were key potential confounding variables measured and adjusted statistically for their impact on the relationship between exposure(s) and outcome(s)?	Quality rating (good, fair, poor)
Mutlu 2011^(38)^	Yes	Yes	NR	Yes	No	No	Yes	Yes	Yes	No	Yes	NR	NA	No	Fair

**Table 5:** Quality assessment tool for before-after (pre-post) studies with no control group^(33)^

	Was the study question or objective clearly stated?	Were eligibility/selection criteria for the study population pre-specified and clearly described?	Were the participants in the study representative of those who would be eligible for the test/service/intervention in the general or clinical population of interest?	Were all eligible participants that met the pre-specified entry criteria enrolled?	Was the sample size sufficiently large to provide confidence in the findings?	Was the test/service/intervention clearly described and delivered consistently across the study population?	Were the outcome measures pre-specified, clearly defined, valid, reliable, and assessed consistently across all study participants?	Were the people assessing the outcomes blinded to the participants’ exposures/interventions?	Was the loss to follow-up after baseline 20% or less? Were those lost to follow-up accounted for in the analysis?	Did the statistical methods examine changes in outcome measures from before to after the intervention? Were statistical tests done that provided *P*-values for the pre-to-post-changes?	Were outcome measures of interest taken multiple times before the intervention and multiple times after the intervention (i.e. did they use an interrupted time series design)?	If the intervention was conducted at a group level (e.g. a whole hospital, a community, etc.), did the statistical analysis take into account the use of individual-level data to determine effects at the group level?	Quality rating (good, fair, poor)
Ganmaa *et al.* (2008)^(39)^	Yes	No	No	Yes	Yes	Yes	Yes	NR	Yes	Yes	No	Yes	Fair
Rajakumar *et al.* (2008)^(40)^	Yes	Yes	Yes	NR	Yes	Yes	Yes	NR	Yes	Yes	No	Yes	Good
Rajakumar *et al.* (2005)^(41)^	Yes	Yes	Yes	NR	Yes	Yes	Yes	No	Yes	Yes	No	Yes	Good

### Behavioural interventions

All studies included conducted a vitamin D pharmacological supplementation intervention in our target population, with two that incorporated a behavioural component to their intervention strategy^(38,42)^ in addition to the pharmacological intervention. We found no study that explicitly defined a primary aim of evaluating a behavioural intervention undertaken with the intention to study its effect on vitamin D supplement uptake.

Madar *et al.*^(42)^ (Table 2; overall risk of bias: some concerns) studied the effect of vitamin D_2_ drops on serum 25-hydroxy-vitamin D (25(OH)D) in infants with immigrant origin within a cluster RCT. In total, sixty-six healthy infants of Pakistani (South Asian origin), Turkish (West Asian/Middle Eastern) or Somali (East African) origin were recruited for the study from eight child health clinics in Oslo, Norway. The behavioural component involved multilingual translated brochures for mothers, with information provided to each ethnic group on vitamin D, its sources and instructions on how to administer the vitamin D drops, made available free of charge. Aims were to evaluate whether a free supply of a 400 iu daily dose, for 6-week old infants, together with information handouts that had been translated into Urdu, Turkish or Somali languages incorporating text and simple illustrations, improved vitamin D status, assessed at 7-week follow-up in the intervention and control group. Fifty-one (78 %) infants completed the study, with serum 25(OH)D levels significantly higher in the intervention group *v*. Control group (93·5 *v*. 72·7 nmol/l; *P* = 0·03). Amongst exclusively breast-fed infants at baseline, serum 25(OH)D levels increased by 32·3 nmol (*P* = 0·035) in the intervention group. This study concluded^(42)^ that free supply of vitamin D drops, with translated information handouts, significantly improved the vitamin D status of healthy infants of immigrant background. Considering the Behaviour Change Wheel framework, this combined intervention strategy (if part of an explicitly stated behavioural intervention) could have been coded under criteria; education and training (translated information leaflets; administration advice) and enablement (free supply)^(30)^.

The second study with behavioural components centred around a nationwide prevention campaign instigated due to resurgence of vitamin D deficiency rickets in children of ethnic minority origin living in Turkey, a sunny country in West Asia/Middle East. In order to gauge the campaign’s impact which included supply of vitamin D supplements as an intervention, Mutlu *et al.*^(38)^ (Table 4; overall risk of bias: Fair) report from evaluation undertaken with a small sample of 85 healthy infants (45 girls and 40 boys; individual ethnic origin data not provided). The over-arching aims for the campaign, which also incorporated a curriculum to train healthcare workers, were to encourage the entire population, and especially pregnant and nursing women and infants, to have adequate sunlight exposure. Distribution of vitamin D supplements to every newborn at no cost to families was made through Turkey’s network of primary care units and maternal health centres. The study had limitations; no control group, high risk of bias from confounding and no statistical test was performed (Table 4). Evaluation was by way of face-to-face interviews with the mothers, advised to administer three drops (400 iu) daily. Overall, seventy-six (89 %) infants received the vitamin D dose recommended. However, mothers for twenty-six (31 %) infants stated that they did not administer the dose on a daily basis. The research team concluded that for prevention of vitamin D deficiency in infants, a daily dose of 400 iu was sufficient. Considering the Behaviour Change Wheel framework, the intervention strategy utilised could be coded under criteria of education and enablement^(30)^. The authors^(38)^ noted that the major obstacles for use of vitamin D supplements in Turkey included limited public awareness, access to healthcare and supplement costs.

### Pharmacological interventions

All studies included in this review incorporated some form of pharmacological intervention, the results of which are summarised in the following sections. Ten studies^(7,37,38,42–48)^ were undertaken during the pregnancy/newborn phase with ethnic minority origin as a main risk factor. One study specifically targeted a cohort with known ‘at-risk status’, that is, nutritional rickets^(44)^.

### Pregnancy/newborn group

Green *et al.*’s^(37)^ observational study assessed sixty-five Canadian breast-fed infants (Asian/White; aged 0·2–0·4 weeks), noting that supplementation with 10 mcg vitamin D helped infants achieve significantly higher 25(OH)D concentrations compared with those who received less (mean 25(OH)D 9·4 ng/ml higher; *P* = 0·003). Maternal vitamin 25(OH)D levels, season, skin colour and ethnicity were not significant determinants of infant vitamin 25(OH)D levels. Hanson *et al.*’s study^(46)^ also undertaken in Canada evaluated newborn infants receiving formula feeds during intensive care hospitalisation. Classified as White or non-White African American origin, the study arms included supplementation with 400 iu vitamin D or matching placebo with formula feeds. White infants achieved significantly higher mean 25(OH)D levels by time of discharge (*P* = 0·0003).

Hollis *et al.* reported findings from two studies^(43,48)^. The 2011 US cohort^(48)^ were women with singleton pregnancies, given varying doses of vitamin D (400/2000/4000 iu daily) from 12–16 weeks of gestation to delivery. The 4000 iu daily dose was safe and the most effective in achieving sufficiency in all women regardless of race (Caucasian; African American; Hispanic; *P* < 0·0001). The 2015 US study^(43)^ included 334 mother–infant pairs with newborns of 4–6 weeks old, recruited as mothers planned to breast-feed for 6 months. Compared to a 400 iu daily dose, the higher 6400 iu daily dose showed a significant increase in maternal vitamin D levels (*P* < 0·0001) and was safe. Vitamin D deficiency in breast-fed infants was affected by race, with African American mothers and infants having substantially lower circulating 25(OH)D levels.

Rodda *et al.*’s Australian study^(45)^ recruited 78 ethnic minority pregnant women and newborns, identified on the basis of dark skin or veiling (84 % treated and 97 % control group). They were supplemented with 2000–4000 iu daily from 12 to 16 weeks’ gestation to delivery. Umbilical cord serum 25(OH)D levels at delivery were higher in neonates of mothers in the treated group (81 nmol/l *v*. 42 nmol/l; *P* < 0·0001).

Yu *et al.*’s prospective UK study^(7)^ recruited 180 women, of Indian Asian, Middle Eastern, Black and Caucasian origin, treated from 27 weeks’ gestation to delivery. Study arms compared a single 200 000 iu dose injection, an 800 iu daily dose and no treatment. Treated mothers had significantly improved vitamin D levels (42 *v*. 27 nmol/l; *P* < 0·0001), but only a small percentage (women 30 %; babies 8 %) given supplements achieved sufficiency levels (50 nmol/l or over).

The intervention in Hossain *et al.*’s single-centre open-label RCT^(47)^ in Pakistan (South Asia) was 4000 iu vitamin D taken daily from gestational week 20 to delivery. Positive correlation was found between maternal and neonatal 25(OH)D levels (*r* = 0·83; *P* = 0·001), with improved status with supplementation (maternal 25(OH)D increased from mean 8·82 ng/dl to 18·3 ng/dl; *P* = 0·001). Mondal *et al.*^(44)^ studied seventy-one Indian children (South Asia) with nutritional rickets, aged between 6 months and 5 years, given either a single intramuscular dose of 600 000 iu vitamin D or 10 weeks of a 60 000 iu dose taken weekly. Both were safe and effective with no difference found in efficacy on comparing diagnostic parameters for rickets (*P* > 0·05).

The Madar *et al.*^(42)^ and Mutlu *et al.*^(38)^ studies discussed above included a behavioural component in addition to the pharmacological intervention.

### Children

Mondal *et al.*’s^(44)^ study on children aged between 0·5 and 5 years is discussed above. Of fourteen further papers in children (aged 1–20 years), ten were undertaken in the USA and included African American^(40,41,49–52)^, Hispanic, Asian and White^(36)^, or Black and White^(53–55)^ children. None reported including any type of behavioural intervention.

Lewis *et al.*^(54)^ assessed five daily oral vitamin D doses (0,400,1000,2000 and 4000 iu) in Black and White children aged 9–13 years. Increases in vitamin 25(OH)D levels depended on dose taken; 12-week changes ranged between −10 and 76 nmol/l (placebo *v*. 4000 iu, respectively). In the highest dose group, larger vitamin D gains were seen in White compared with Black children (*P* < 0·01)

Rajakumar *et al.* reported vitamin D insufficiency at baseline^(41)^ in about half of their study group (6–10 years old, African American children). Using the 2005 definition for adults, deficiency status was defined as less than 10 ng/ml and insufficiency as between 10 and 20 ng/ml^(41)^. Supplementation with 400 iu daily for 4 weeks helped achieve a significant increase in levels (see Table 1), but insufficiency status still persisted in 18 % of the experimental group^(41)^. In a separate study, Rajakumar *et al.* also noted^(40)^ that vitamin D deficiency is common amongst obese and non-obese preadolescent African American children, finding that 400 iu daily dose was not enough to raise vitamin D serum levels to 30 ng/ml (Table 1; deficiency defined as less than 20 ng/ml; insufficiency 21–29 ng/ml). In further work^(49,52)^, Rajakumar *et al.* studied African American and White children aged 8–14 years supplemented with vitamin D_3_ 1000 iu/d for 6 months. They reported that vitamin D_3_ supplementation varied with race and was more effective and significant in Black children^(49)^ (Table 1). In addition, the intake required to maintain concentrations at 20 ng/ml in 97·5 % of Black and White children, adjusting for race and pubertal status, was three times higher than the US recommended allowance of 600 iu/d^(52)^. Sachek *et al.*’s^(53)^ randomised control trial recruited 604 children (Black, Hispanic, White and Asian), aged 8–15 years. They found that serum vitamin D levels increased over 6 months with all dose ranges (600, 1000 or 2000 iu daily) with the 2000 iu daily dose achieving a higher vitamin D concentration compared with the two lower doses (33·1 *v*. 26·3 and 27·5 ng/ml; *P* < 0·001). Talib *et al.*^(55)^ found that adolescents (Hispanic, Black, White and Asian; 13–20 years old) required 8 weeks of high-dose colecalciferol, of at least 5000 iu/d in order to correct deficiency status, noting also that obese adolescents had poorer response to treatment (13·7 +/− 10·7 *v*. 21·9 +/− 16·9 ng/ml; *P* < 0·001).

Abrams *et al.*^(36)^ found no significant effect of an increase in vitamin D3, with decrease in parathyroid hormone levels, on calcium absorption, which was one of their study’s primary outcomes of interest. The pharmacological intervention used was supplementation with 1000 iu daily vitamin D for 8 weeks in African American, Hispanic, Asian, and White children aged between 4 and 9 years. Dong *et al.*^(50)^ studied supplementation in forty-nine healthy African American children, aged 14–18 years, with 2000 iu/d *v*. 400 iu/d in the control group. They reported the 2000 iu daily dose as probably more effective (see Table 1) in optimising vitamin D status and counteracting progression of aortic stiffness in Black youth.

Doherty *et al.*’s^(51)^ target group were African American children and young adults (5–20 years old) with or without sickle cell disease (SCD-SS). The study aim was to assess safety and efficacy of two oral vitamin D_3_ daily doses (4000 iu and 7000 iu). They noted that with 12 weeks of supplementation, both doses were safe and well tolerated but deficiency status (<20 ng/ml) was eliminated only in those receiving 7000 iu/d (*P* < 0·05).

Andersen *et al.*^(56)^ recruited adolescent girls of Pakistani (South Asia) origin living in Denmark, aged 10–15 years, to study two vitamin D doses (10, 20 mcg/d). It is difficult to comment on any significant difference in 25(OH)D levels in the group after 1 year, as numbers recruited were small (*n* 21). Ganmaa *et al.*^(39,57)^ found that drinking ultra-high-temperature (UHT) processed milk fortified with about 100 iu vitamin D per serving improved levels in Mongolian (East Asia) children aged 9–11 years, and that correction of baseline deficiency in Mongolian children aged 12–15 years with 800 iu daily over 6 months increased growth^(57)^. Thatcher *et al.*^(58)^ studied Nigerian (West Africa) children aged between 1 and 14 years with nutritional rickets, finding that calcium supplementation with or without vitamin D was more effective then vitamin D alone in healing active rickets (*P* < 0·001). (Tables 1, 6 and 7).

**Table 6:** Pharmacological vitamin D doses used in the trials included in systematic review and intervention outcome

Reference	Age (years)	Vitamin D supplement doses given and frequency	Intervention outcome positive/negative/other	Other study outcomes
Abrams *et al.* (2013)^(36)^	4–9	1000 iu daily for 8 weeks	Positive	No significant effects of change in vitamin D3 level on calcium absorption
Andersen *et al.* 2008^(56)^	10–15	10 mcg or 20 mcg/d dose over 1 year	Positive	Sample included women (18–53 years) and men 18–64 years).
Dong *et al.* (2010)^(50)^	14–18	2000 iu v. 400 iu in control group over 16 weeks	Positive	2000 iu daily maybe effective in optimising vitamin D status and counteracting progression of aortic stiffness in Black youth. Plasma 25(OH)D concentrations negatively modulated by adiposity
Dougherty 2015^(51)^	5–20	4000 iu/d v. 7000 iu/d over 12 weeks	Positive	In both groups of patients with sickle cell disease (SCD-SS), deficiency status eliminated only in those on 7000 iu/d dose.
Ganmaa *et al.* (2008)^(39)^	9–11	Fortified whole milk with 100 iu vitamin D per serving, over 1 month	Positive	
Ganmaa 2017^(57)^	(i) 12–15 (ii) 9–11	(i) 800 iu/d for 6 months (ii) 710-ml whole milk fortified with 310 iu vitamin D taken daily over 7 weeks	Positive	Correcting vitamin D deficiency status in children with 800 iu daily increased growth
Green *et al.* (2015)^(37)^	0·2–0·4	10 mcg vitamin D/dose	Positive	Maternal vitamin 25(OH)D levels, season, skin colour and ethnicity were not significant determinants of infant vitamin 25(OH)D levels.
Hanson *et al.* (2011)^(46)^	Newborn	400 iu daily in addition to formula feed	Positive	White infants had statistically significant higher mean 25(OH)D levels at discharge then non-White infants.
Hollis *et al.* (2011)^(48)^	Pregnant women and newborns	Pregnant women on 400 iu vitamin D with additional 0 iu, 1600 iu or 3600 iu until delivery	Positive	4000 iu/d for pregnant women safe and most effective in achieving sufficiency in all neonates as well regardless of race
Hollis *et al.* (2015)^(43)^	Newborn 4–6 weeks with breast-feeding planned for 6 months	6400 iu vitamin D/d v. 400 iu/d	Positive	6400 iu vitamin D dose daily was safe. Vitamin D deficiency in breast-fed infants affected by race.
Hossain *et al.* 2014^(47)^	Pregnant women and newborns	4000 iu/d from week 20 to delivery	Positive	There was a positive correlation between maternal and neonatal 25(OH)D levels.
Lewis *et al.* (2013)^(54)^	9–13	5 oral doses tested over 12 weeks; 0, 400 iu, 1000 iu, 2000 iu, 4000 iu.	Positive	Larger gains in vitamin D levels in White v. Black population at the highest dose
Madar *et al.* (2009)^(42)^	7 weeks old	400 iu vitamin D_2_ daily over 7 weeks	Positive	Translated information brochures and free supply of vitamin D supplement.
Mondal *et al.* (2014)^(44)^	0·5–5	Single i/m dose of 600 000 iu or 60 000 iu taken weekly for 10 weeks.	Positive	No difference found in efficacy of both regimens when comparing diagnostic parameters of rickets at 12 weeks.
Mutlu 2011^(38)^	Infants (3–120-d-old) recalled as part of national screening; interviews with mothers	Babies provided with vitamin D solution supplement; recommended dose 3 drops daily (400 iu).	Positive	Mothers provided with free vitamin D solution supplement.
Rajakumar *et al.* (2005)^(41)^	6–10	400 iu/d over 4 weeks	Positive	Vitamin D insufficiency status persisted in 18 % study group of African American children
Rajakumar *et al.* (2008)^(40)^	6–10	400 iu/d over 4 weeks	Positive	Dose inadequate to raise blood levels to sufficiency status in both obese and non-obese African American children
Rajakumar *et al.* (2015)^(49)^	8–14	1000 iu/d over 6 months	Positive	Effect of supplementation varied by race and was more effective and significant in Black children
Rajakumar *et al.* (2016)^(52)^	8–14	1000 iu/d over 6 months	Positive	Vitamin D dose required for sufficiency status in 97·5 % of Black and White children threefold higher than recommended RDA 600 iu/d.
Rodda *et al.* (2015)^(45)^	Pregnant women and newborns	2000–4000 iu/d until 28-week gestation	Positive	Umbilical cord serum 25(OH)D concentration at delivery higher in neonates of treatment group mothers.
Sacheck *et al.* (2017)^(53)^	8–15	600 iu, 1000 iu or 2000 iu/d over 6 months	Positive	The 2000 iu/d group achieved a higher vitamin D concentration than the other two dose groups
Talib *et al.* (2016)^(55)^	Adolescents	50 000 u/week or 5000 iu/d or 1000 iu/d over 8 weeks	Positive	Obese adolescents have poorer response to treatment
Thacher *et al.* (1999)^(58)^	1–14	600 000 iu i/m or calcium 1000 mg/d or combined calcium and vitamin D over 24 weeks	Negative	Calcium with or without vitamin D was more effective than supplementation with vitamin D alone in healing active rickets
Yu *et al.* 2009^(7)^	Pregnant women and newborns	Single 200 000 iu dose or 800 iu/d dose or no treatment from 27 weeks gestation to delivery.	Positive	Of those taking supplements, only 30 % women and 8 % babies achieved sufficiency status.

**Table 7:** Vitamin D intervention study in healthy v. at-risk population as defined by research group

References	Author defined study group	Ethnic group study	Classification of vitamin D status in study
Dong *et al.* (2010)^(50)^	Healthy group:		
	Healthy Study of two doses of vitamin D	Yes	Sufficiency: 75 nmol/l Insufficiency: more than 50 but less than 75 nmol/l Deficiency: under 50 nmol/l)
Abrams *et al.* (2013)^(36)^	Healthy	Yes	High 32 ng/ml; 50–80 nmol/l Middle 2–32 ng/ml; 50–80 nmol/l Low 20 ng/ml; 50 nmol/l
	Vitamin D at-risk group:		
Thacher *et al.* (1999)^(58)^	Rickets or bone deformities	Nigerian children, in Nigeria	
Rajakumar *et al.* (2005)^(41)^	At-risk vitamin D sample	Yes	(i) 12–20 ng/ml essential for bone health in children (ii) Insufficiency: 10–20 ng/ml Deficiency: < 10 ng/ml based on definition for adults
Rajakumar *et al.* (2008)^(40)^	Obese African American	Yes	Insufficiency: 21–29 ng/ml deficiency: < 20 ng/ml
Mondal *et al.* (2014)^(44)^	Nutritional rickets	Indian children, in India	
Dougherty 2015^(51)^	Sickle cell disease Study of two doses of vitamin D	Yes	Suboptimal vitamin D status (<32 ng/ml) Deficiency 20 g/ml
Talib *et al.* (2016)^(55)^	Adolescents with vitamin D deficiency	Yes	
	Pregnancy/neonate:		
Yu *et al.* 2009^(7)^	Pregnancy	Yes	Sufficient > 50 nmol/l Insufficient 25–50 nmol/l Deficiency < 25 nmol/l
Madar *et al.* (2009)^(42)^	Neonate	Yes	
Hollis *et al.* (2011)^(48)^	Pregnancy/newborn	Yes	Sufficiency ≥80 nmol/l (≥32 ng/ml) Insufficiency as ≥50 to <80 nmol/l (≥20 to <32 ng/ml) Deficiency <50 nmol/l (20 ng/ml)
Hanson *et al.* (2011)^(46)^	Infants/formula fed	Yes	
Mutlu 2011^(38)^	Infants	Turkish infants; part of Turkey’s nationwide prevention programme.	
Hossain *et al.* 2014^(47)^	Pregnancy/neonate	Pakistani women and infants in Pakistan	
Hollis *et al.* (2015)^(43)^	Mother–infant pairs and breast-feeding	Yes	
Green *et al.* (2015)^(37)^	Breast-fed infants	Yes	
Rodda *et al.* (2015)^(45)^	Pregnancy/newborn vitamin D-deficient	Yes	Deficiency or insufficiency: <75 nmol/l
	Other:		
Andersen *et al.* 2008^(56)^	Study of two doses of vitamin D	Yes	
Ganmaa *et al.* (2008)^(39)^	Fortification	Mongolian children, in Mongolia	
Lewis *et al.* (2013)^(54)^	Study of five oral doses in children	Yes	
Rajakumar *et al.* (2015)^(49)^	Study of 1000 iu daily dose for 6 months	Yes	
Rajakumar *et al.* (2016)^(52)^	Study of 1000 iu daily dose for 6 months	Yes	
Ganmaa 2017^(57)^	Correction of baseline deficiency; fortification	Mongolian children, in Mongolia	
Sacheck *et al.* (2017)^(53)^	Study of three doses of vitamin D	Yes	

### Research published after completion of the initial review

The updated literature review on MEDLINE, EMBASE, CINAHL and Cochrane Library databases, including secondary search of Google Scholar, undertaken for February 2018–November 2020, focused purely on identifying behavioural intervention studies. No publication with an explicitly defined behavioural intervention implemented to study vitamin D supplementation outcomes was identified (see Fig. 1). Protocols for two new systematic reviews have been published^(66,67)^, but there is no indication that any behavioural interventions per se will be considered.

## Discussion

This systematic review included twenty-four publications for final evaluation. We had planned to analyse behavioural interventions using HBM constructs^(28)^ and the Behaviour Change Wheel framework criteria^(30)^. We have utilised HBM constructs for assessment in a previous patient and public involvement study^(29)^.

Despite our a priori aim, we found no studies that explicitly defined and studied the effects of a behavioural intervention. Therefore, we completed a narrative evaluation of the two studies that undertook a pharmacological intervention and incorporated a behavioural component, which included translated information brochures, some support from health professionals and free supply of vitamin D supplements^(38,42)^. These studies did not evaluate which individual component helped improve vitamin D status in the infants, for example, whether it was the translated information brochure provided to the mothers or the free supplement supply. The combined strategies broadly meet criteria of education, training and enablement from the total of nine that underpin the Behaviour Change Wheel model^(30)^.

In general, there was a great deal of variability in the approaches of the studies. Several studies were undertaken in healthy populations^(36,42,50,54)^, including populations where the aim was to evaluate a large-scale national prevention campaign^(38)^ or study the effect of fortification^(39,57)^. Other studies were undertaken in patients with medical conditions that precluded achievement of vitamin D sufficiency status (Table 7). Examples include a sickle cell anaemia – SS patient cohort^(51)^, infants in intensive care^(46)^, treatment for rickets^(44,58)^ or studying treatment in obesity^(40,53)^. Variation in study outcomes is to be expected due to heterogeneity within the studies, which used different thresholds for insufficiency and deficiency status when measuring serum vitamin D levels^(7,36,40,41,50,57)^. Hanson *et al.*^(46)^, Rodda *et al.*^(45)^ and Yu *et al.*^(7)^ undertook cord blood sampling, whereas Hollis *et al.*^(43)^ evaluated maternal and infant urine and blood samples. The majority of the studies aimed to assess whether a vitamin D 400 iu daily dose was sufficient, using various surrogate endpoints to rationalise and justify their conclusions, with study of higher doses in studies that included patients with research group-defined deficiency/insufficiency status (Tables 1 and 7).

Cochrane reviews have shown that supplements taken during pregnancy could reduce the risks for pre-eclampsia, gestational diabetes and low birth weight^(9,59)^. The 2018 Food Standards Agency’s commissioned research on use of food supplements^(60)^ found that consumers with higher levels of education or currently working were more likely to take supplements. The most common reason cited for taking food supplements was an aspiration for a healthy lifestyle. However, consumers who may be at risk may not be adequately informed about food supplements, to include vitamin D. Michie and Tayarachakul^(61)^ suggest that any ‘information and advisory’ health professional role for pregnant women should include the recommendation of 400 iu vitamin D daily; with communication, especially with vulnerable mothers, and these should include mothers of darker skinned ethnic populations, undertaken and supported by midwives or community pharmacists^(61)^. High-risk groups may need individualised advice and information on the need for vitamin D supplements. In addition, it may be important to address perceived medicalisation of vitamin D supplements, whereby women feel they only need to take a supplement on the recommendation or prescription from their GP^(29)^. Currently in the UK, patients and the public are advised to purchase vitamin D, unless eligible for the Healthy Start Scheme^(19)^. Even if prescribed per se, there are important issues that need to be addressed. Wan *et al.*^(62)^ demonstrated an increase in prescribing of vitamin D in the UK, but their 2008–2016 database study found inconsistency between supply regimens prescribed, an absence of pre-supplementation (range 29–56 % annually), and a trend for increased prescribing of higher pharmacological treatment doses rather than maintenance doses. Global and national data^(1–9,11,12,16–18,20,59–61,63)^ show there are still ongoing issues relating to vitamin D deficiency status and supplementation. More importantly, studies have not adequately addressed the best approach to improve uptake in high-risk groups. This area requires further work to identify the most effective behavioural interventions, which should ideally be co-developed working with the high-risk community itself.

We used ethnicity as an inclusion criterion in order to consider at-risk groups of Asian or African origin. Both culture and religion can impact on uptake of supplements, but no study in this review discussed this complex relationship and effect on uptake, compliance or adherence^(64,65)^ with vitamin D supplementation. We noted variations in study design to support improved uptake of pharmacological treatment and these observations could be used as supporting information for future behavioural interventions. These modifications included use of chewable tablets^(53)^, liquid^(37,51)^ or flavoured formulations^(36,53)^, drops for infants^(37,38,42,51)^ and halal-certified products^(45,56)^. Some of these modifications would be applicable in the case of all children, whereas some are important when considering ethnicity and culturally acceptable treatment.

The Palacios *et al.*^(9)^ updated Cochrane ‘interventions’ systematic review aimed to evaluate the effect of vitamin D supplementation. With a focus on pregnancy only and not on at-risk ethnic minority groups per se, they assessed the evidence base for three distinct patient cohorts; vitamin D only (22 trials; 3725 pregnant women), calcium and vitamin D supplementation (9 trials; 1916 pregnant women) and vitamin D and other micronutrients (1 study; 1300 pregnant women). The studies considered pharmacological interventions evaluating risk or harm; no behavioural interventions were described. As in our review, they observed that supplementation increased serum 25(OH)D concentrations during pregnancy but with large heterogeneity in the results, possibly related to differences in vitamin D doses and methods used to assess outcomes. Another Cochrane review^(59)^ considered variations in vitamin D supplement regimens during pregnancy, focusing on pregnancy and neonatal outcomes, but did not specifically consider at-risk ethnic minority groups and no behavioural interventions were reported.

Although we identified few publications in this systematic review, there is work that shows that milk fortification^(35,53)^ appears to be acceptable to mothers, and chewable formulations^(53)^ in addition to drops or liquid products^(34,35,39,47)^ appear tolerable in children. This could have important implications for public policy on mass fortification. Variations in the available forms of supplements should be highlighted, especially to those groups less likely to present to health services.

Structural barriers will be important to overcome to ensure messaging reaches at-risk groups, including health literacy and low socio-economic status, especially where the targeted community comprises first-generation immigrants or residents. Other facets that deserve due consideration include individual communication preferences, comprehension and concerns, and research teams will need to seriously consider these with use of co-production methodologies. The NIHR INVOLVE guidance^(24)^ defines the approach used by researchers, practitioners and the public working together on a co-produced research project as one of joint ownership, with sharing of power and responsibility from the start to the end of the project. Working together helps promote understanding by including all perspectives and skills. With close community engagement, there is an expectation that implementation of the co-designed intervention will result in better outcomes.

The two behavioural type strategies that we identified used elements of education, training and enablement as possible supportive behaviour change techniques, with the associated pharmacological intervention demonstrating achievement of significantly higher vitamin D levels. As these were mainly medical intervention trials, it is to be expected that the focus would be on participant adherence rather than on changing behaviour related to ongoing supplementation. However, bearing in mind racial/ethnic disparities, using a behavioural strategy may be important. We accepted that the search term ‘interventions’ would identify both pharmacological- and behavioural-type interventions, and we evaluated on the premise that there can be learning from any behavioural component incorporated within pharmacological trials as well. Future research groups could consider incorporating explicitly defined behavioural interventions, underpinned by HBM constructs and Behaviour Change Wheel categories, as a formal aspect of their intervention study. This may help address structural barriers and could be implemented, for example, during routine antenatal visits, for widespread population benefits. It is important to note the results of a 2020 study of 125 UK children with nutritional rickets, which found that over three-quarters (77·6 %) of these children were not taking vitamin D supplements^(2)^. Accepting that there is still a need for more data on children who did not have nutritional rickets, it is evident that current recommendations from Department of Health^(1,3,19)^ are not reaching high-risk groups. Our aim to study behavioural interventions aiming to improve both initiation and maintenance of supplementation in pregnancy and beyond has been informed by this observation. The findings of our study, namely a lack of research into behavioural interventions and the forms of intervention that would be most effective in engaging at-risk groups, lead us to recommend this as a research priority.

### Limitations of this review

Mutlu *et al.*’s study^(38)^ describes a national campaign implemented in Turkey, aiming to encourage the entire population, including pregnant and nursing women and infants, to have adequate sunlight exposure. Although the primary source of vitamin D is sun exposure, the ability of the body to create and maintain sufficient levels is affected by many variables. These include socio-economic/socio-demographic status, geographical/environmental, and cultural and religious, lifestyle and dietary as well as genetic factors. Knoss *et al.*^(68)^ studied people of different ethnic origins with similar practices, noting that skin pigmentation was a significant risk factor for vitamin D deficiency, irrespective of ultra-violet light exposure. A Swedish, primarily a food-based intervention study^(69)^, did not report ethnic origin but recruited children living in the northern and southern latitudes of the country. With children stratified by skin colour using Fitzpatrick’s definition, the results showed that those with darker skin required higher vitamin D intake or supplementation. We focused on ethnicity to help identify behavioural interventions implemented, if any, but note that there may be other relevant work on sun exposure-related behavioural interventions in children. We used ethnicity as a primary search term to help identify high-risk groups, but studies reported their selected populations based on ethnic origin or alternatively used race for classification. This adds to the heterogeneity of data available. No studies considered inter-related complexities including cultural or religious beliefs, distance from country of origin, or first- or second-generation immigrant status, and this needs due acknowledgement. The primary focus for all studies was outcome of a pharmacological intervention, rather than behavioural interventions, with different thresholds used to define vitamin D deficiency and extrapolation of findings should be undertaken cautiously. We only included studies published in English, and studies where full text was available, meaning some relevant studies may have been omitted.

## Conclusions

In summary, all the studies included in our systematic review, which focused on high-risk ethnic groups aged under 18 years, evaluated the effect of a pharmacological vitamin D intervention. There were no studies identified that included direct evaluation of an explicitly defined behavioural intervention. There is a need for additional rigorous high-quality and larger RCT to evaluate the effects of vitamin D supplementation in pregnancy. Equally, there is a need for research into clearly defined behavioural interventions targeted to individual ethnic groups, ideally designed with use of co-production methodologies, in order to help our understanding of how behaviour change can affect vitamin D use in antenatal care, pregnancy and childhood.

## Supplementary material

For supplementary material accompanying this paper visit https://doi.org/10.1017/S1368980021000756. click here to view supplementary material
