# Supplementary material for: Interventions to improve vitamin D status in at-risk ethnic groups during pregnancy and early childhood: a systematic review
Source: Public Health Nutr. 2021 Feb 17;24(11):3498–519. doi: 10.1017/S1368980021000756 (PMC10195367; doi:10.1017/S1368980021000756)
Supplement: Supplementary file 1 [file S1368980021000756sup001.docx]

**Updated search and review: February 2018- November 2020.**

1. Total screened by Librarian: 619 and additional Cochrane library search abstracts

Embase  46 of 281 selected by librarian for further review; Medline 25 of 85;

CINHAL - after eliminating duplicates……...49 of 253 articles

1. Total titles and abstracts independently reviewed by RP and NT: 120

- Confirmed with EA for nil ‘behavioural’ interventions’ studies: 100

**MEDLINE**

1. *VITAMINS/ OR *CALCITRIOL/
2. (vitamin d).ti,ab
3. [search not used]
4. ("child* at risk").ti,ab
5. (ethnic minorities).ti,ab
6. (ethnic group*).ti,ab
7. (1 OR 2)
8. (5 OR 6)
9. (4 AND 8)
10. (7 AND 9)
11. *"EARLY MEDICAL INTERVENTION"/
12. *"PREVENTIVE HEALTH SERVICES"/ OR *"CHILD HEALTH SERVICES"/
13. (11 OR 12)
14. (2 AND 13)
15. (7 AND 8 AND 13)
16. (2 AND 4)
17. (13 AND 16)
18. (2 AND 8)
19. (13 AND 18)
20. (birth AND vitamin d).ti,ab
21. (8 AND 20)
22. (4 OR 5 OR 6)
23. (20 AND 22)

44. (optimisation OR intervention).ti,ab
45. *"VITAMIN D DEFICIENCY"/ OR *RICKETS/
46. *"25-HYDROXYVITAMIN D 2"/
47. (vitamin d).ti,ab
48. (45 OR 46 OR 47)
49. *"INFANT, NEWBORN"/
50. (newborn OR child).ti,ab
51. (49 OR 50)
52. (48 AND 51)
53. (44 AND 52)

**EMBASE**

1. (vitamin d).ti,ab 93848
2. *"VITAMIN D"/ OR *"25 HYDROXYVITAMIN D"/
3. ("deficiency of vitamin d").ti,ab
4. (child*).ti,ab
5. ("ethnic minorities").ti,ab
6. ("early intervention").ti,ab
7. (24 OR 25)
8. (26 AND 30)
9. (27 AND 28)
10. (31 AND 32)
11. (26 AND 29)
12. (27 AND 30)
13. (26 AND 35)
14. *INFANT/ OR *NEWBORN/
15. (newborn OR child*).ti,ab
16. (37 OR 38)
17. (30 AND 39)
18. (26 AND 40)
19. (28 AND 41)
20. (29 AND 41)

54. (optimisation OR intervention).ti,ab
55. *"VITAMIN D DEFICIENCY"/ OR *RICKETS/
56. *"25-HYDROXYVITAMIN D 2"/
62. *"INFANT, NEWBORN"/
65. (newborn OR child).ti,ab
66. (vitamin d).ti,ab
67. *"VITAMIN D"/
68. (55 OR 56 OR 66 OR 67)
69. *"EARLY INTERVENTION"/
70. (54 OR 69)
71. (68 AND 70)
72. (62 OR 65)

73. (71 AND 72)

**CINAHL**

74. (vitamin d).ti,ab

75. *"VITAMIN D DEFICIENCY"/

76. (child*).ti,ab

77. *INFANT/ OR *CHILD/

78. *"ETHNIC GROUPS"/

79. (ethnic minorit*).ti,ab

80. *"INFANT, NEWBORN, DISEASES"/

81. (newborn).ti,ab

82. (prevention).ti,ab

83. ("early intervention").ti,ab

84. *"EARLY CHILDHOOD INTERVENTION"/

85. (early intervention).ti,ab

86. *"EARLY INTERVENTION"/

87. (74 OR 75)

88. (76 OR 77 OR 80 OR 81)

89. (78 OR 79)

90. (82 OR 83 OR 84 OR 85 OR 86)

91. (87 AND 88)

92. (90 AND 91) 253

93. (89 AND 92)

**Cochrane Systematic Review Database:**

Search Keywords: "child deficiency and vitamin D".
